# Supplementary material for: CASZ1 induces skeletal muscle and rhabdomyosarcoma differentiation through a feed-forward loop with MYOD and MYOG
Source: Nat Commun. 2020 Feb 14;11:911. doi: 10.1038/s41467-020-14684-4 (PMC7021771; doi:10.1038/s41467-020-14684-4)
Supplement: Supplementary file 1 — Supplementary Information [file 41467_2020_14684_MOESM1_ESM.pdf]

Supplementary Information

CASZ1 induces skeletal muscle and embryonal rhabdomyosarcoma differentiation via formation of a feed-forward differentiation loop with MYOD and MYOG

Liu et al.

Supplementary Figure 1

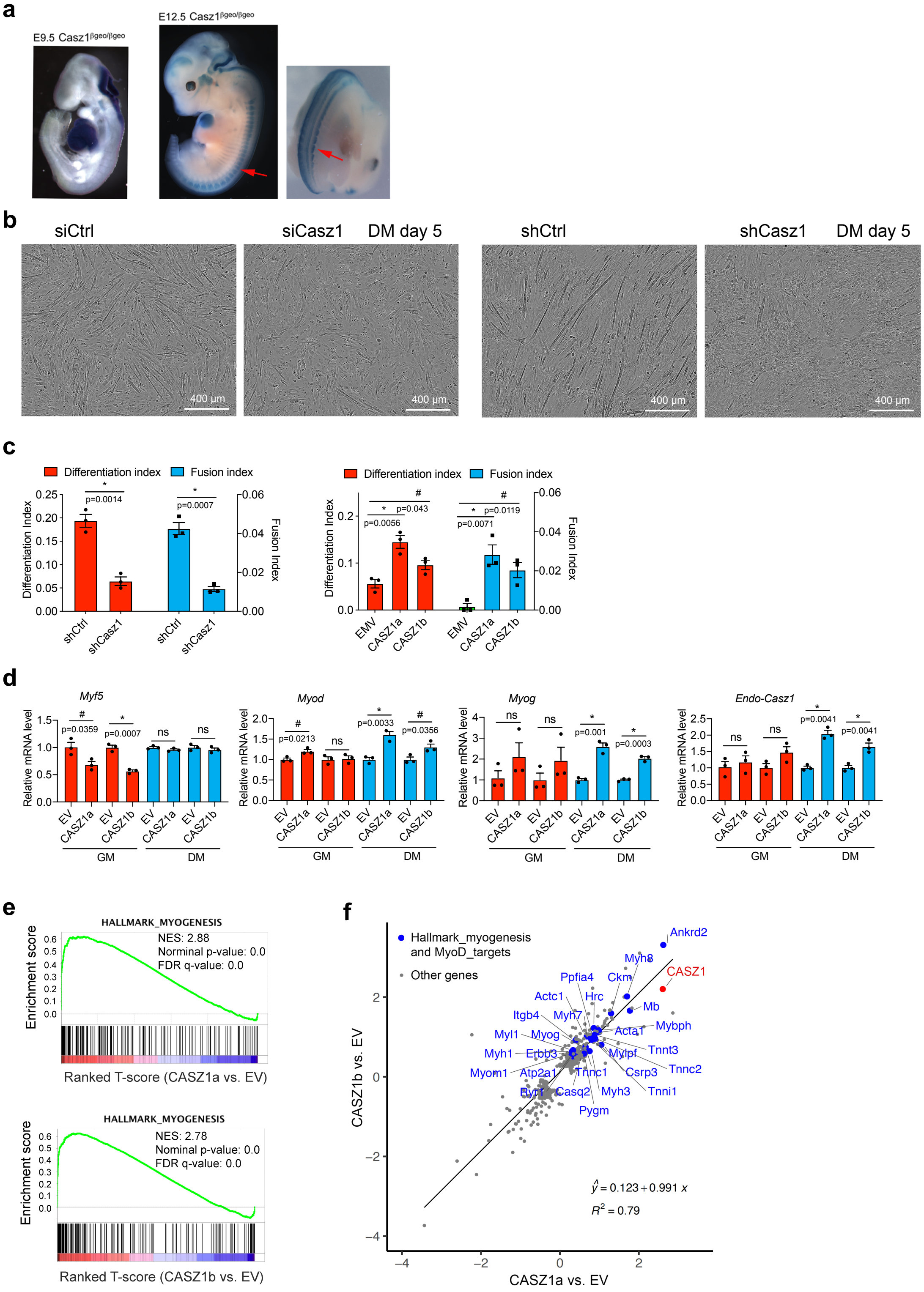

Supplementary Figure 1. Casz1 regulates skeletal muscle differentiation

a, In Casz1 trapped mouse embryo model, the beta-geo reporter was under the control of endogenous CASZ1 promoter, beta-gal staining showed that Casz1 expression is detectable in the somites of E12.5

(red arrow) but not E9.5 mouse embryo. **b**, Knockdown of Casz1 using siRNA (left panel) or shRNA (right panel) attenuates myotube formation induced by DM (bright field). **c**, Knockdown Casz1 using shRNA in C2C12 cells attenuates muscle differentiation induced by DM as assessed by a decrease in differentiation and fusion indices compared to shCtrl (left panel); overexpression of either human CASZ1a or CASZ1b accelerates muscle differentiation as shown by an increase differentiation and fusion indices compared to EV (right panel). **d,** Either human CASZ1a or CASZ1b is overexpressed in C2C12 cells and cultured in GM for 48 hr; or cultured in GM for 24 hr then switched to DM for another 24 hr, RT-PCR is performed to investigate the MRFs and endogenous Casz1 mRNA levels. Overexpression of CASZ1a or CASZ1b significantly decreases Myf5 mRNA level compared to EV in GM condition; overexpression of CASZ1a or CASZ1b significantly increases MyoD, Myog and endogenous Casz1 (Endo_Casz1) mRNA levels compared to EV in DM condition. **e**, GSEA shows the positive enrichment of myogenesis genes in C2C12 cells that over-expressed CASZ1a (top panel) or over-expressed CASZ1b (bottom panel). **f,** Changes in expression of genes regulated by CASZ1a (CASZ1a vs. EV) and CASZ1b (CASZ1b vs. EV) are significantly-positively correlated based on RNA-seq results. Data represent mean ± SEM, n = 3 biological replicates, ns not significant. Two-sided Student's *t*-test was used to calculate statistical difference. Source data are provided as a Source Data file.

Supplementary Figure 2

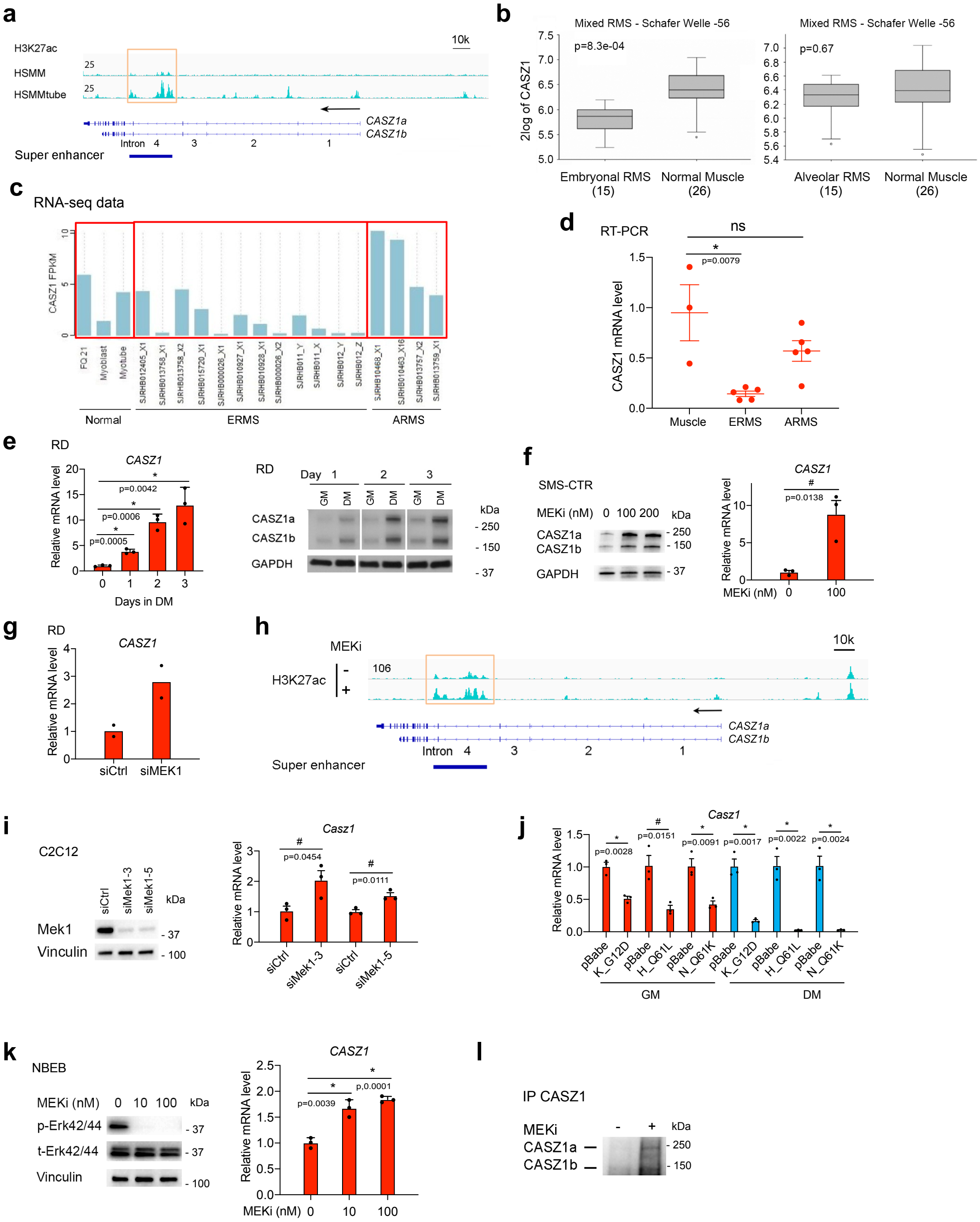

m

Physiological System Development and Function

Name	p-value
Embryonic Development	1.44E-06 - 1.46E-20
Organismal Development	1.44E-06 - 1.46E-20
Nervous System Development and Function	1.44E-06 - 3.37E-19
Organ Development	1.44E-06 - 1.04E-18
Skeletal and Muscular System Development and Function	1.08E-06 - 1.04E-18

Supplementary Figure 2. The regulation of CASZ1.

a, CASZ1 is driven by a SE in human skeletal muscle myotubes. **b**, Schafer Welle RMS microarray dataset show that CASZ1 mRNA levels are lower in ERMS compared to normal muscle. Data are presented as box and whisker plots with middle lines indicating medians and whiskers representing the 25th and 75th percentiles. The graph is generated by R2 database. **c,** St. Jude RMS dataset show that CASZ1 mRNA levels are lower in ERMS compared to ARMS and normal muscle (FQ21: fetal quadriceps). **d,** RT-PCR on three normal skeletal muscle samples, five ERMS and five ARMS patient samples show that mRNA levels of CASZ1 are significantly lower in ERMS compared to normal skeletal muscle. **e,** CASZ1 mRNA levels and protein levels (right panel) are upregulated when RD cells are cultured in differentiation medium (100 nM TPA). **f**, Western blot results and RT-PCR results show that both CASZ1 protein levels and mRNA levels are increased when SMS-CTR cells are treated with MEKi. **g**, Knockdown of MEK1 using siRNA in RD cells increases CASZ1 mRNA levels as determined by RT-PCR. **h,** There is an acquisition of SE in CASZ1 gene loci in MEKi-induced, differentiated SMS-CTR cells. **i,** The knockdown of Mek1 using Mek1 siRNAs in C2C12 cells results in a decrease in Mek1 protein levels detected by western blot and an increase of Casz1 mRNA levels detected by RT-PCR. **j,** Over-expression of mutant KRAS (K_G12D), HRAS (H_Q61L) or NRAS (N_Q61K) in C2C12 cells decreases *CASZ1* mRNA levels in either GM or DM. **k,** MEKi treatment of NBEB neuroblastoma cells results in a decrease of phospho-ERK42/44 detected by western blot and an increase of CASZ1 mRNA levels detected by RT-PCR. **l,** Immunoprecipitation of CASZ1 using anti-CASZ1 antibody from formaldehyde cross-linked control or MEKi-induced, differentiated SMS-CTR cells showed the pull-down of CASZ1a and CASZ1b. **m,** IPA CASZ1 binding site-associated genes in MEKi-induced, differentiated SMS-CTR cells. Data represent mean, n = 2 biological replicates for g; Data represent mean ± SEM, n = 3 biological replicates, ns not significant for the rest panels. Two-sided Student's *t*-test was used to calculate statistical difference. Source data are provided as a Source Data file.

Supplementary Figure 3

a CASZ1 and MYOD common binding sites

Physiological System Development and Function

Name	p-value range
Embryonic Development	2.60E-06 - 9.83E-19
Organismal Development	2.88E-06 - 9.83E-19
Organ Development	2.33E-06 - 4.51E-18
Skeletal and Muscular System Development and Function	2.48E-06 - 4.51E-18
Tissue Development	2.48E-06 - 4.51E-18

b MYOD specific binding sites

Physiological System Development and Function

Name	p-value range
Organismal Development	1.45E-08 - 1.37E-24
Embryonic Development	4.96E-09 - 3.49E-24
Organ Development	1.53E-08 - 3.49E-24
Skeletal and Muscular System Development and Function	1.24E-08 - 3.49E-24
Tissue Development	1.73E-08 - 3.49E-24

c CASZ1 specific binding sites

Physiological System Development and Function

Name	p-value range
Nervous System Development and Function	5.04E-04 - 7.34E-11
Cardiovascular System Development and Function	5.22E-04 - 7.38E-11
Embryonic Development	4.82E-04 - 7.81E-11
Organismal Development	4.82E-04 - 7.81E-11
Connective Tissue Development and Function	4.50E-04 - 2.32E-10

d **e**

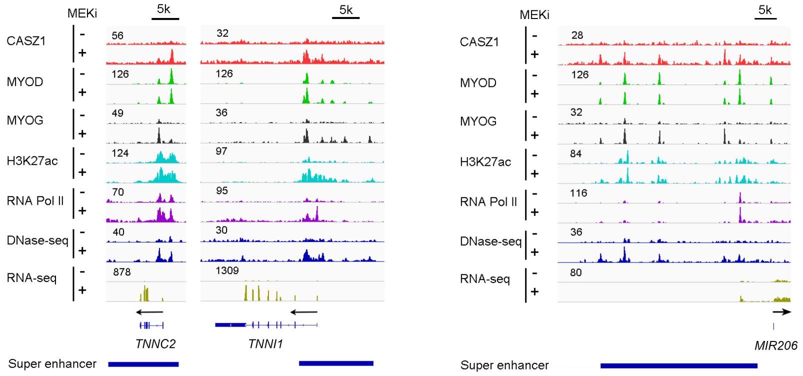

Supplementary Figure 3. ChIP-seq analysis of CASZ1 in differentiated SMS-CTR cells
a-c, Ingenuity pathway analysis shows that CASZ1 & MYOD common binding sites or MYOD specific binding site-associated genes are enriched in skeletal and muscular system development (ranked as top 4), but CASZ1 specific binding site-associated genes are enriched in nervous system development and function (ranked as top 1). **d**, Signal tracks show the binding and co-occupancy of CASZ1, MYOD, MYOG, H3K27ac, RNA Pol II on skeletal muscle genes *TNNC2* and *TNNI1* before (-) and after (+) MEKi treatment. **e**, Signal tracks show the binding and co-occupancy of CASZ1, MYOD, MYOG, H3K27ac, RNA Pol II on microRNA MIR206 gene locus in SMS-CTR cells before (-) and after (+) MEKi induced differentiation.

Supplementary Figure 4

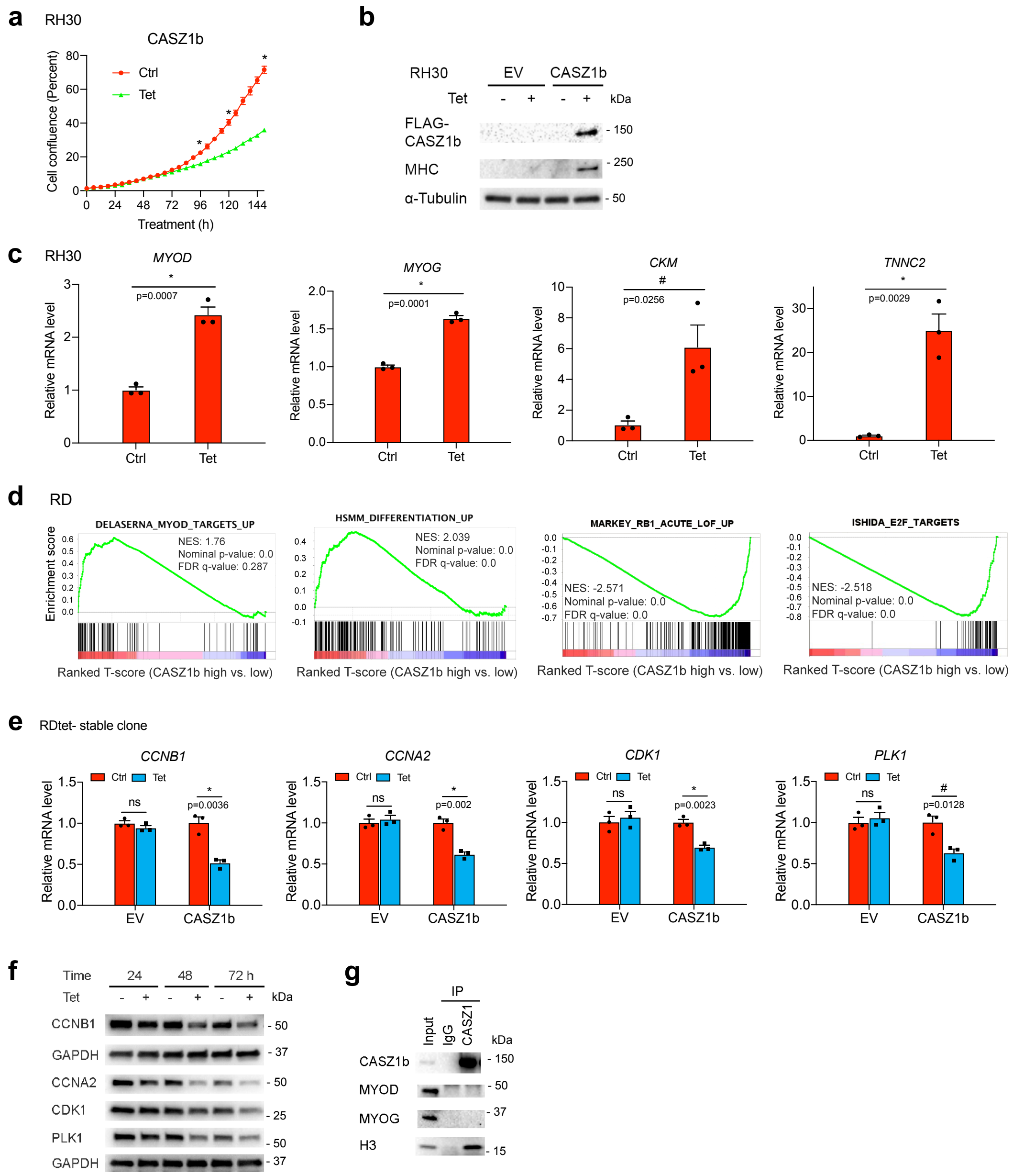

Supplementary Figure 4. Restoration of CASZ1b in RMS cells induces cell differentiation
a, FLAG tagged CASZ1b is stably cloned into ARMS (RH30) cells and CASZ1b expression is Tetracycline (Tet) inducible. Restoration of CASZ1b in RH30 cells (Tet treatment) inhibits cell proliferation compared to control cells (Ctrl) as shown by an IncuCyte cell confluence assay. **b**, Restoration of CASZ1b in RH30 cells increases expression of the skeletal muscle differentiation marker, MHC, protein detected by western blot. **c,** RT-PCR results show that two days induction of CASZ1b by Tet treatment leads to a decrease of the mRNA levels of myogenic regulatory factors and skeletal muscle differentiation markers. **d**, GSEA shows that restoration of CASZ1b in RD cells leads to a positive enrichment of MYOD signature genes (Here the FDR is 0.287, it is slightly over the threshold of significance of 0.25), skeletal muscle differentiation genes; and a negative enrichment of pRB repressed genes and E2F target genes. **e**, RT-PCR results show that two days induction of CASZ1b by Tet treatment leads to a decrease of the mRNA levels of cell cycle genes. Here empty vector (EV) transfected cells

serve as a control to evaluate Tet effect. **f**, Western blot shows that CASZ1b represses genes required for cell cycle progression at protein levels. **g**, Co-immunoprecipitation in CASZ1b restored SMS-CTR cells using anti-CASZ1 antibody showed the pulling down of a known CASZ1b protein partner histone 3 (H3), but not MYOD or MYOG. Data represent mean ± SEM, n = 3 biological replicates, ns not significant. Two-sided Student's *t*-test was used to calculate statistical difference. Source data are provided as a Source Data file.

Supplementary Figure 5

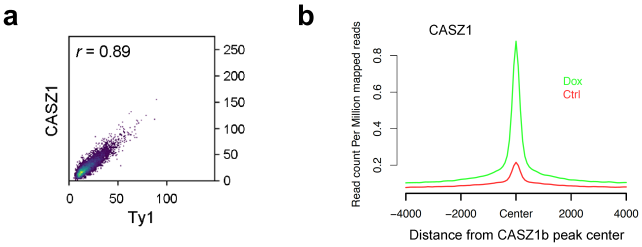

c

CASZ1 HOMER *de novo* motif			
**Rank**	**Motif**	**P-value**	**Best Match**
**1**	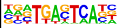	**1e-1860**	**FRA1, FOSL2 JUNB, AP-1**
**2**	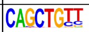	**1e-766**	**MYOD, MYOG MYF5, TCF12**

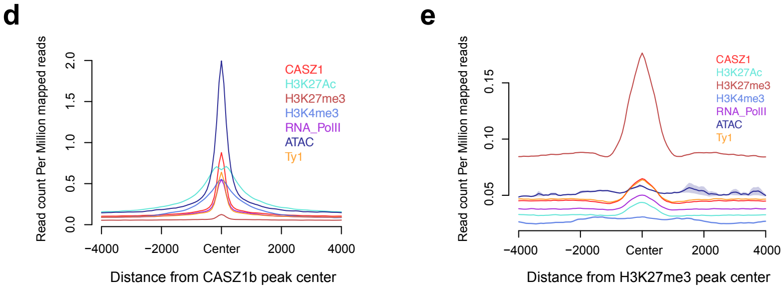

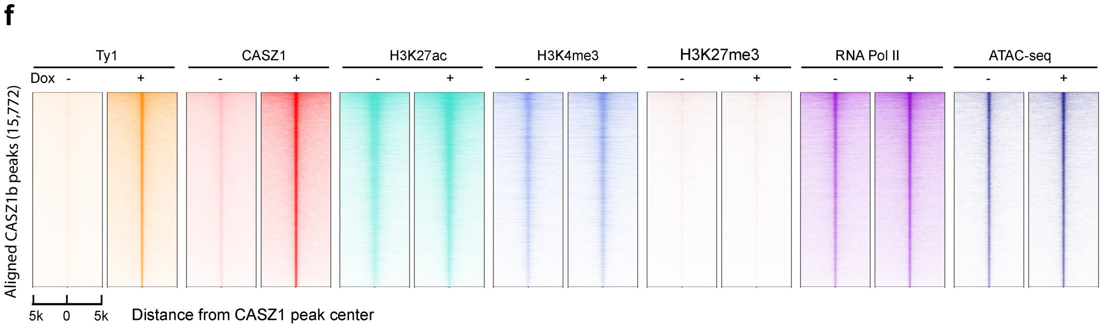

g

CASZ1b directly up-regulated genes

Physiological System Development and Function

Name	p-value range
**Cardiovascular System Development and Function**	2.85E-03 - 3.42E-08
**Organ Morphology**	2.48E-03 - 1.63E-07
**Organismal Development**	2.85E-03 - 1.63E-07
**Skeletal and Muscular System Development and Function**	2.85E-03 - 2.50E-06
**Tissue Development**	2.85E-03 - 3.31E-06

h

CASZ1b directly down-regulated genes

Physiological System Development and Function

Name	p-value range
**Cardiovascular System Development and Function**	4.70E-06 - 2.05E-23
**Organismal Development**	4.88E-06 - 2.05E-23
**Tissue Development**	4.88E-06 - 5.58E-19
**Nervous System Development and Function**	2.65E-06 - 2.95E-15
**Tissue Morphology**	2.24E-06 - 3.30E-13

i

IPA annotation	P-Value	Z-score
Cell movement of smooth muscle cells	1.82E-07	-3.24
Proliferation of smooth muscle cells	9.06E-07	-2.05
Migration of muscle cells	1.21E-06	-3.09
Formation of cartilage tissue	1.33E-06	-2.00
Migration of smooth muscle cells	1.78E-06	-3.09

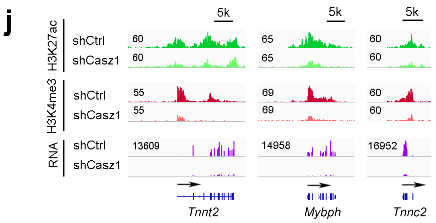

Supplementary Figure 5. ChIP-seq analysis of CASZ1b when it is restored in SMS-CTR cells
a, In 3xTy1 tagged CASZ1b restored SMS-CTR cells, ChIP-seq data got from anti-Ty1 antibody and anti-CASZ1 antibody shows significant Pearson correlation. **b**, Composite plot shows the increase of CASZ1b binding on genomic DNA after CASZ1b restored in SMS-CTR cells (Dox) for 48 hr. **c**, Homer de novo motif scan of CASZ1b binding peaks shows the enrichment of AP1 binding motifs and MRFs binding motifs. **d**, Composite plot shows the co-occupancy of Ty1 (anti-Ty1 antibody to pull-down Ty1-CASZ1b), H3K27ac, H3K4me3, RNA Pol II, ATAC-seq but not H3K27me3 at CASZ1b peak center. **e**, Composite plot shows that there is almost no co-occupancy of CASZ1b, H3K27ac, H3K4me3, RNA Pol II and ATAC-seq signal at H3K27me3 peak center. **f**, Heatmap shows the ranked CASZ1b binding peaks (15,772) and the aligned peaks of Ty1, H3K27ac, H3K4me3, RNA Pol II, ATAC-seq at CASZ1b peak center before (-) and after (+) Dox treatment. **g**, In SMS-CTR cells, CASZ1b directly up-regulated genes are involved in skeletal muscular system development and function (ranked as top 4). **h,** CASZ1b directly down-regulated genes are involved in nervous system development and function (ranked as top 4). **i,** When focused on skeletal muscular system development and function of CASZ1b directly down-regulated genes, they are involved in muscle cell movement. **j**, Signal tracks show that the knockdown of Casz1 in C2C12 cells led to a decrease of H3K27ac and H3K4me3 signals and transcriptional activation of skeletal muscle genes induced by culturing cells in DM.

Supplementary Figure 6

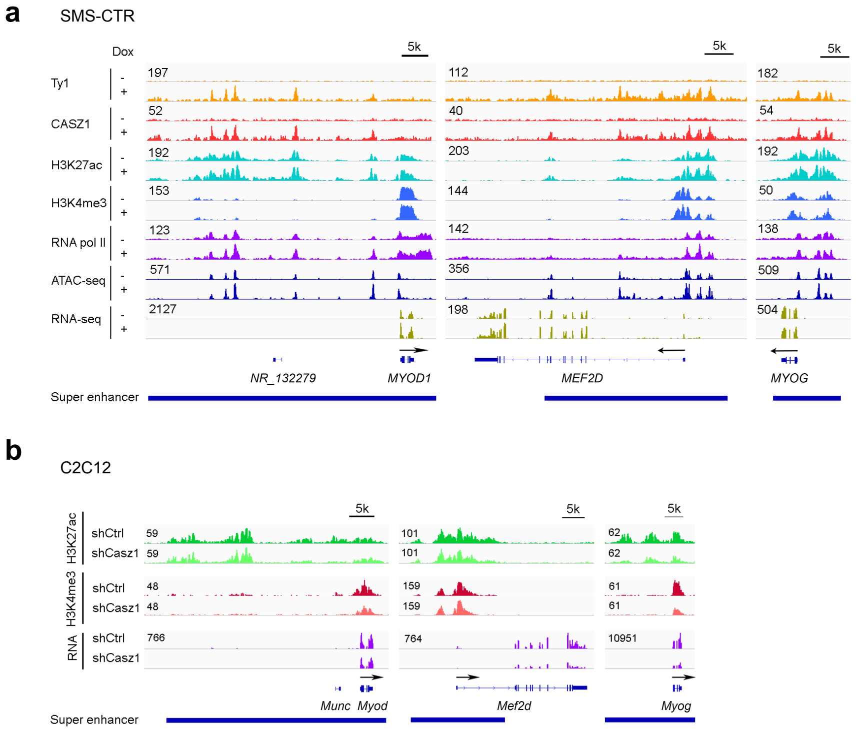

Supplementary Figure 6. CASZ1b affects SE signal on MRFs when it is restored in SMS-CTR cells
a, Signal tracks show that in CASZ1b restored SMS-CTR cells, the SE signal that drives MYOD1, MEF2D and MYOG is increased. **b**, Signal tracks show that when Casz1 was knocked down in C2C12 cells (DM), the SE signal that drives MYOD, MEF2D and MYOG is decreased.

Supplementary Figure 7

a

Nucleotide Change	Amino acid Change	Frequency in RMS (%)	Dbsnp137	NHLBI6500 ALL	NHLBI6500 EA	nci60	cg69	RMS Subtype	SIFT Prediction	SIFT Score	PolyPhen2 Prediction	PolyPhen2 Probability
C73T	R25C	1.18	rs200934862	0.0158	0.0238	0	0	ERMS	Damaging	0	Deleterious	1
G969C	E323D	1.18	0	0	0	0	0	ARMS	Damaging	0	Deleterious	0.996
G2026T	G676C	1.18	0	0	0	0	0	ARMS	Damaging	0	Deleterious	1
T3386C	M1129T	1.18	0	0	0	0	0	ERMS	Tolerated	1	Deleterious	0.763

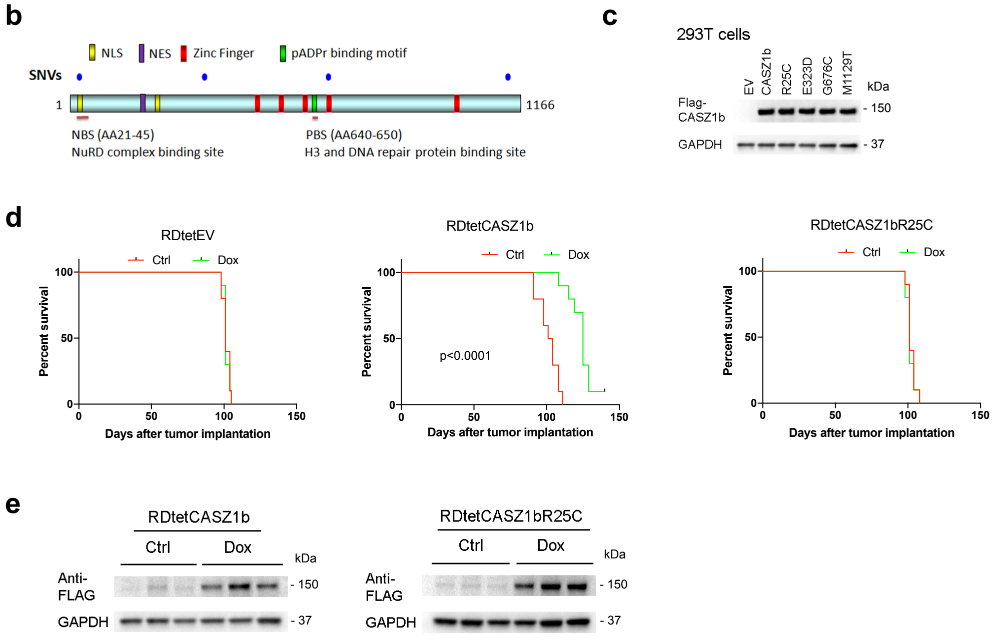

Supplementary Figure 7. CASZ1 SNVs identified in RMS patients

a, Information of CASZ1 SNVs. **b**, Schematic diagram shows SNVs localization on CASZ1b protein. **c**, Western blot shows the transient overexpression of CASZ1b and mutant constructs in HEK293T cells. **d**, Dox alone has no effect on murine survival probability in empty vector (EV) transfected ERMS cells generated xenografts (left panel). Mice with wild-type CASZ1b but not CASZ1bR25C xenografts have prolonged survival (middle and right panel). **e**, Western blot shows the induction of CASZ1b and CASZ1bR25C protein in the tumors from the xenografts. The log-rank (Mantel-Cox) test was used to compare event-free survival distributions between treatment groups. Source data are provided as a Source Data file.
