# Supplementary material for: CASZ1 induces skeletal muscle and rhabdomyosarcoma differentiation through a feed-forward loop with MYOD and MYOG
Source: Nat Commun. 2020 Feb 14;11:911. doi: 10.1038/s41467-020-14684-4 (PMC7021771; doi:10.1038/s41467-020-14684-4)
Supplement: Supplementary file 3 — Description of Additional Supplementary Files [file 41467_2020_14684_MOESM3_ESM.pdf]

Description of Additional Supplementary Files

File Name: Supplementary Data 1
Description: MyoD and Myog ChIP-seq peaks identified through publicly available ChIP-seq data (GSE44824) analysis.

File Name: Supplementary Data 2
Description: The whole RNA-seq gene list in C2C12 cells after knockdown of Casz1 or over-expression of CASZ1a or CASZ1b.

File Name: Supplementary Data 3
Description: The comparison of changes in expression of genes significantly regulated by CASZ1a or CASZ1b after increased of either isoform in C2C12 cells.

File Name: Supplementary Data 4
Description: CASZ1 bound peaks identified by ChIP-seq in MEKi induced, differentiated SMS-CTR cells.

File Name: Supplementary Data 5
Description: The comparison of CASZ1 bound peaks and MYOD bound peaks identified by ChIP-seq in MEKi induced, differentiated SMS-CTR cells.

File Name: Supplementary Data 6
Description: The whole RNA-seq gene list in RDtetCASZ1b cells or SMS-CTRtetCASZ1b cells after turning on CASZ1b.

File Name: Supplementary Data 7
Description: Important gene signatures regulated by CASZ1b after restoration in SMS-CTR cells based on RNA-seq and GSEA.

File Name: Supplementary Data 8
Description: Top 100 gene sets regulated by CASZ1b in C2C12 myoblasts and SMS-CTR cells (ERMS) after 48 hr overexpression of CASZ1b identified through RNA-seq and GSEA.

File Name: Supplementary Data 9
Description: CASZ1b bound peaks identified using anti-CASZ1 antibody or anti Ty1 antibody in SMS-CTRtetCASZ1b cells that treated with or without Dox.

File Name: Supplementary Data 10
Description: The list of genes that directly bound and transcriptionally regulated by CASZ1b in SMS-CTRtetCASZ1b cells after turning on the expression CASZ1b.

File Name: Supplementary Data 11
Description: Skeletal muscle genes that directly bound and transcriptionally regulated by CASZ1b in SMS-CTR cells based on ChIP-seq and RNA-seq analysis.

File Name: Supplementary Data 12
Description: List of super-enhancers before and after turning on CASZ1b in SMS CTRtetCASZ1b cells.
