# Supplementary material for: CASZ1 induces skeletal muscle and rhabdomyosarcoma differentiation through a feed-forward loop with MYOD and MYOG
Source: Nat Commun. 2020 Feb 14;11:911. doi: 10.1038/s41467-020-14684-4 (PMC7021771; doi:10.1038/s41467-020-14684-4)
Supplement: Supplementary file 16 — Reporting Summary [file 41467_2020_14684_MOESM16_ESM.pdf]

nature research

Corresponding author(s): Zhihui Liu & Carol J. Thiele

Last updated by author(s): Dec 16, 2019

Reporting Summary

Nature Research wishes to improve the reproducibility of the work that we publish. This form provides structure for consistency and transparency in reporting. For further information on Nature Research policies, see Authors & Referees and the Editorial Policy Checklist.

Statistics

For all statistical analyses, confirm that the following items are present in the figure legend, table legend, main text, or Methods section.

n/a	Confirmed	
☐	☒	The exact sample size (*n*) for each experimental group/condition, given as a discrete number and unit of measurement
☐	☒	A statement on whether measurements were taken from distinct samples or whether the same sample was measured repeatedly
☐	☒	The statistical test(s) used AND whether they are one- or two-sided<br>*Only common tests should be described solely by name; describe more complex techniques in the Methods section.*
☐	☒	A description of all covariates tested
☐	☒	A description of any assumptions or corrections, such as tests of normality and adjustment for multiple comparisons
☐	☒	A full description of the statistical parameters including central tendency (e.g. means) or other basic estimates (e.g. regression coefficient) AND variation (e.g. standard deviation) or associated estimates of uncertainty (e.g. confidence intervals)
☐	☒	For null hypothesis testing, the test statistic (e.g. *F*, *t*, *r*) with confidence intervals, effect sizes, degrees of freedom and *P* value noted<br>*Give P values as exact values whenever suitable.*
☒	☐	For Bayesian analysis, information on the choice of priors and Markov chain Monte Carlo settings
☒	☐	For hierarchical and complex designs, identification of the appropriate level for tests and full reporting of outcomes
☐	☒	Estimates of effect sizes (e.g. Cohen's *d*, Pearson's *r*), indicating how they were calculated

Our web collection on statistics for biologists contains articles on many of the points above.

Software and code

Policy information about availability of computer code

Data collection	Encode atac_dnase_pipelines was used for ATAC-seq data collection and analysis; trimmomatic 0.39 and Partek Flow was used for RNA-seq data collection and analysis. ChIP-seq data collection and analysis is shown in ChIP-seq reporting summary. The software and tools are installed on NIH Biowulf.
Data analysis	ComplexHeatmap package in R 3.4.3<br>Partek Flow<br>Parteck Genomics Suite v7.17<br>DESeq2<br>QIAGEN's Ingenuity® Pathway Analysis (IPA®, QIAGEN)<br>GraphPad Prism 8.1.0<br>Microsoft Excel 2019<br>Microsoft Word 2019<br>R version 3.5.2 with standard packages<br>picard/2.18.27<br>R/3.5<br>R/3.5.2<br>bwa/0.7.10<br>samtools/1.2<br>igvtools 2.4.16<br>homer/4.10.1<br>fastqc/0.11.8<br>macs/2.1.2<br>bedtools 2.27.1<br>deeptools 3.1.3<br>ROSE

bamliquidator/1.3.4
meme/5.0.1
ngsplot/2.63
python 2.7
ChIPpeakAnno 3.16.1; ComplexHeatmap 1.20.0; cluster 2.0.7; dendextend 1.10.0;
GenomicRanges 1.34.0; plyr 1.8.4
Adobe Illustrator CC 2017
Adobe Photoshop CC 2017
R2 database (2017)

For manuscripts utilizing custom algorithms or software that are central to the research but not yet described in published literature, software must be made available to editors/reviewers. We strongly encourage code deposition in a community repository (e.g. GitHub). See the Nature Research guidelines for submitting code & software for further information.

Data

Policy information about availability of data

All manuscripts must include a data availability statement. This statement should provide the following information, where applicable:

- Accession codes, unique identifiers, or web links for publicly available datasets
- A list of figures that have associated raw data
- A description of any restrictions on data availability

All the home generated RNA-seq, ChIP-seq and ATAC-seq datasets, the GEO accession number is GSE126147.
Link:
https://www.ncbi.nlm.nih.gov/geo/query/acc.cgi?acc=GSE126147.
Accession key:
abahoaowhtcffix

Public available datasets:

Data set	GEO number	SRX number
Yohe_SMS-CTR_D48_input_rep 2	GSE85169, GSE85171	SRX1998417
Yohe_SMS-CTR_T48_input	GSE85169, GSE85171	SRX1998415
Yohe_SMS-CTR_D48_H3K27ac	GSE85169, GSE85171	SRX1998404
Yohe_SMS-CTR_T48_H3K27ac_rep 2	GSE85169, GSE85171	SRX1998406
Yohe_SMS-CTR_D48_MYOD1_rep 2	GSE85169, GSE85171	SRX1998423
Yohe_SMS-CTR_T48_MYOD1	GSE85169, GSE85171	SRX1998421
Yohe_SMS-CTR_D48_MYOG	GSE85169, GSE85171	SRX1998424
Yohe_SMS-CTR_T48_MYOG	GSE85169, GSE85171	SRX1998425
Yohe_SMS-CTR_D48_PolII	GSE85169, GSE85171	SRX1998426
Yohe_SMS-CTR-T48_PolII	GSE85169, GSE85171	SRX1998427
Yohe_SMS-CTR_Dnase_D48_rep 2	GSE85169, GSE85171	SRX1998392
Yohe_SMS-CTR_Dnase_T48_rep 2	GSE85169, GSE85171	SRX1998395
Yohe_SMS-CTR_D48_RNA-seq	GSE85170, GSE85171	SRX1998431
Yohe_SMS-CTR_T48_100nM_RNA-seq	GSE85170, GSE85171	SRX1998437
Bernstein_HSMM_Input	GSE29611	SRX067414
Bernstein_HSMMtube_Input	GSE29611	SRX067399
Bernstein_HSMM_H3K27ac	GSE29611	SRX067506
Bernstein_HSMMtube_H3K27ac	GSE29611	SRX067417
Blum_C2C12_Input_MB	GSE76010	SRX1557409
Blum_C2C12_Input_MT_rep1	GSE76010	SRX1482290
Blum_C2C12_H3K27ac_MB_Rep1	GSE76010	SRX1482271
Blum_C2C12_H3K27ac_MT_Rep1	GSE76010	SRX1482273
Soleimani_C2C12_MB_Myf5	GSE24852	SRX029143
Marinov_C2C12_MyoD	GSE44824	SRX246932
Marinov_C2C12_Myog	GSE44824	SRX246932

Field-specific reporting

Please select the one below that is the best fit for your research. If you are not sure, read the appropriate sections before making your selection.

☒ Life sciences ☐ Behavioural & social sciences ☐ Ecological, evolutionary & environmental sciences

For a reference copy of the document with all sections, see nature.com/documents/nr-reporting-summary-flat.pdf

Life sciences study design

All studies must disclose on these points even when the disclosure is negative.

Sample size	Sample sizes were chosen based on expected phenotypes and previous experience with assay variability, as well as the minimum number of animals that required to do power calculation.

Data exclusions	No data were excluded from these assays.
Replication	Each experiment was done in replicated. The numbers were indicated in figure legend. For example, for cell proliferation assay, cell differentiation assay were repeated three or more times. RNA-seq experiments were performed in three biological replicates. Moreover, majority of the experiments were also confirmed through using different techniques and using multiple cell lines, such as the regulation of ERMS cell proliferation by CASZ1 was confirmed in two ERMS cell lines; transcriptome regulated by CASZ1 was analyzed in three cell lines; differentially expressed genes identified from RNA-seq was confirmed by realtime PCR; gene expression regulation was validated by both realtime PCR and western blot in different cell lines; the regulation of CASZ1 by RAS-MEK pathway was confirmed by both genetic inhibition and pharmaceutic inhibition of RAS-MEK pathway.
Randomization	N=20 of 4 to 6 weeks old SCID female Beige mice for each cell line were randomly divided in 2 groups: one group of mice received normal chow diet while the other group received Dox-containing chow diet for over 5 days. All mice were injected with 2 x 10^6 cell suspension into the left hind limb at the gastrocnemius muscle of the mice. Each group of the mice continue to receive the same diet as before the cell injection.
Blinding	Blinding was not performed since it was not necessary for this study.

Reporting for specific materials, systems and methods

We require information from authors about some types of materials, experimental systems and methods used in many studies. Here, indicate whether each material, system or method listed is relevant to your study. If you are not sure if a list item applies to your research, read the appropriate section before selecting a response.

Materials & experimental systems

n/a	Involved in the study
☐	☒ Antibodies
☐	☒ Eukaryotic cell lines
☒	☐ Palaeontology
☐	☒ Animals and other organisms
☒	☐ Human research participants
☒	☐ Clinical data

Methods

n/a	Involved in the study
☐	☒ ChIP-seq
☒	☐ Flow cytometry
☒	☐ MRI-based neuroimaging

Antibodies

Antibodies used	Primary antibodies used for immunoblotting (WB) or immunofluoresence staining (IF):<br>CASZ1, home made by collaborating with Rockland Inc, affinity purified, named as Cas-ab8540, validated by siRNA, dilution 1:5000 for WB;<br>FLAG, Sigma-Aldrich, catalog number F1804, lot number SLBJ4607V, dilution 1:1000 for WB, 1:50 for IF;<br>Ty1, Diagenode, catalog number C15200054, lot number 006, dilution 1:4000 for WB;<br>MHC, EMD Millipore, catalog number 05-716, lot number DAM1408805, dilution 1:1000 for WB, 1:50 for IF;<br>MYOD, EMD Millipore, catalog number MAB3878, lot number LV1370583, dilution 1:1000 for WB;<br>MYOG, EMD Millipore, catalog number MAB3876, lot number LV1388792, dilution 1:1000 for WB;<br>α-Tubulin, CST, catalog number 3873s, 1:4000 for WB;<br>Total ERK1/2, CST, catalog number 9102, lot number 20, dilution 1:1000 for WB;<br>phospho-ERK1/2 (T202/Y204), CST, catalog number 9106, lot number 38, dilution 1:1000 for WB;<br>VINCULIN, CST, catalog number 4650s, lot number 2, dilution 1: 2000 for WB;<br>GAPDH, Santa Cruz, catalog number sc-25778, dilution 1:2000 for WB;<br>HRAS, Santa Cruz, catalog number sc-520, dilution 1:1000 for WB;<br>Histone 3, Active Motif, catalog number 39163, dilution 1:3000 for WB,<br><br>Secondary antibodies:<br>Goat anti-mouse IgG Alexa Fluor 594, Molecular Probes, catalog number A-11032, dilution 1:250 for IF;<br>Goat anti-mouse IgG HRP, Santa Cruz, catalog number sc-2005, lot number B2213, dilution 1:2000 for WB;<br>Goat anti-rabbit IgG HRP, Santa Cruz, catalog number sc-2004, lot number B2216, dilution 1:2000 for WB<br><br>ChIP-seq antibodies:<br>CASZ1, home made by collaborating with Rockland Inc, affinity purified, named as Cas-ab8540<br>H3K27ac, Abcam, catalog number ab4729, lot number GR232896-1;<br>H3K27me3, EMD Millipore, catalog number 07-449, lot number 3083046;<br>H3K4me3, CST, catalog number 9751, lot number 10;<br>RNA polymerase II, Active Motif, catalog number 39097, lot number 30617017;<br>Ty1, Diagenode, catalog number C15200054, lot number 006
Validation	Commercially available antibodies used have been validated by their respective manufactures' catalogue files to be compatible. Antibodies for ChIP-Seq including histone marks, RNA Pol II and Ty1 have been validated for ChIP assays by the manufactures and have been used for ChIP-seq by researchers in many publications.

Specificity of antibody against CASZ1 was confirmed by peptide competition (Liu et al., Plos One 2011), transient siRNA against CASZ1 protein, followed by western blotting. To validate CASZ1 antibody for ChIP-seq, we performed immunoprecipitation of formaldehyde cross-linked cells that with low or high CASZ1 expression, followed by western blotting. We also compared the ChIP-seq results of CASZ1b-3xTy1 fusion protein transfected cells using anti-CASZ1 antibody and anti-Ty1 antibody and demonstrated the results are significantly correlated (pearson's correlation=0.89).

Eukaryotic cell lines

Policy information about cell lines

Cell line source(s)

C2C12 and HEK293T cell lines were obtained from ATCC. Rhabdomyosarcoma RD and SMS-CTR cell lines were originally obtained from ATCC, and RH30 was originally obtained from Peter Houghton's lab, where this cell line was established.

Authentication

All cell lines used were STR tested for identity by Cancer Genomics Research Laboratory (CGR) at NCI.

Mycoplasma contamination

All cells were routinely tested for mycoplasma and confirmed negative.

Commonly misidentified lines
(See ICLAC register)

No commonly misidentified cell lines were used in this study.

Animals and other organisms

Policy information about studies involving animals; ARRIVE guidelines recommended for reporting animal research

Laboratory animals

Groups of 4 to 6 weeks old female Fox Chase SCID Beige: CB17.B6-PrkdcscidLyst bg/Crl (SCID Beige) mice were injected with 2 x 10^6 in 100µl cell suspension mixed with matrigel (1:1) into the left hind limb at the gastrocnemius muscle. In all experiments, the leg dimensions were measured twice a week with digital calipers to obtain two diameters of the tumor diameter, from which the tumor volume was determined. All mice were euthanized when any of the tumor diameters were approaching 20 mm in any dimension.

Wild animals

No wild animals were used in this study.

Field-collected samples

No field-collected samples were used in this study.

Ethics oversight

All Xenograft studies were approved by the National Cancer Institute’s Animal Care and Use Committee (ACUC), and all animal care was in accordance with institutional guidelines.

Note that full information on the approval of the study protocol must also be provided in the manuscript.

ChIP-seq

Data deposition

☒ Confirm that both raw and final processed data have been deposited in a public database such as GEO.

☒ Confirm that you have deposited or provided access to graph files (e.g. BED files) for the called peaks.

Data access links
May remain private before publication.

GEO accession number for data generated in this study is GSE126147.
Link:
https://www.ncbi.nlm.nih.gov/geo/query/acc.cgi?acc=GSE126147.
Accession key:
abahoaowhtcffix

Files in database submission

GSM number	processed data file	raw file
GSM3592269	CTRtetCASZ1b_Ty1_Ctrl.bw	CTRtetCASZ1b_Ty1_Ctrl.fastq.gz
GSM3592270	CTRtetCASZ1b_Ty1_Dox.bw	CTRtetCASZ1b_Ty1_Dox.fastq.gz
GSM3592271	CTRtetCASZ1b_CASZ1_Ctrl.bw	CTRtetCASZ1b_CASZ1_Ctrl.fastq.gz
GSM3592272	CTRtetCASZ1b_CASZ1_Dox.bw	CTRtetCASZ1b_CASZ1_Dox.fastq.gz
GSM3592273	CTRtetCASZ1b_H3K27Ac_Ctrl.bw	CTRtetCASZ1b_H3K27Ac_Ctrl.fastq.gz
GSM3592274	CTRtetCASZ1b_H3K27Ac_Dox.bw	CTRtetCASZ1b_H3K27Ac_Dox.fastq.gz
GSM3592275	CTRtetCASZ1b_H3K27me3_Ctrl.bw	CTRtetCASZ1b_H3K27me3_Ctrl.fastq.gz
GSM3592276	CTRtetCASZ1b_H3K27me3_Dox.bw	CTRtetCASZ1b_H3K27me3_Dox.fastq.gz
GSM3592277	CTRtetCASZ1b_H3K4me3_Ctrl.bw	CTRtetCASZ1b_H3K4me3_Ctrl.fastq.gz
GSM3592278	CTRtetCASZ1b_H3K4me3_Dox.bw	CTRtetCASZ1b_H3K4me3_Dox.fastq.gz
GSM3592279	CTRtetCASZ1b_RNA_PolII_Ctrl.bw	CTRtetCASZ1b_RNA_PolII_Ctrl.fastq.gz
GSM3592280	CTRtetCASZ1b_RNA_PolII_Dox.bw	CTRtetCASZ1b_RNA_PolII_Dox.fastq.gz
GSM3592281	CTRtetCASZ1b_Input_Ctrl.bw	CTRtetCASZ1b_Input_Ctrl.fastq.gz
GSM3592282	CTRtetCASZ1b_Input_Dox.bw	CTRtetCASZ1b_Input_Dox.fastq.gz
GSM3592283	SMS-CTR_DMSO_CASZ1.bw	SMS-CTR_DMSO_CASZ1.fastq.gz
GSM3592284	SMS-CTR_Trametinib_CASZ1.bw	SMS-CTR_Trametinib_CASZ1.fastq.gz
GSM3592285	SMS-CTR_DMSO_Input.bw	SMS-CTR_DMSO_Input.fastq.gz
GSM3592286	SMS-CTR_Trametinib_Input.bw	SMS-CTR_Trametinib_Input.fastq.gz

GSM3592263	C2C12_H3K27Ac_DM_shCtrl.bw	C2C12_H3K27Ac_DM_shCtrl.fastq.gz
GSM3592264	C2C12_H3K27Ac_DM_shCas.bw	C2C12_H3K27Ac_DM_shCas.fastq.gz
GSM3592265	C2C12_H3K4me3_DM_shCtrl.bw	C2C12_H3K4me3_DM_shCtrl.fastq.gz
GSM3592266	C2C12_H3K4me3_DM_shCas.bw	C2C12_H3K4me3_DM_shCas.fastq.gz
GSM3592267	C2C12_Input_shCtrl.bw	C2C12_Input_shCtrl.fastq.gz
GSM3592268	C2C12_Input_shCas.bw	C2C12_Input_shCas.fastq.gz

Genome browser session
(e.g. UCSC)

N/A

Methodology

Replicates

For each experiment, 4-5 individual 15 cm dishes of control or treatment group of C2C12 cells or SMS-CTR cells were fixed and cell pellet were collected from each dish. 4-5 pellets were sonicated per group and sonicated nuclear extracts were pooled, mixed well and aliquoted. 20 - 50 μg sonicated chromatin was used for each ChIP.

Sequencing depth

ChIP-sequencing was performed using Illumina NextSeq single end 75bp reads.

Sample	Total Reads	Mapped Reads
SMS-CTRtetCASZ1_Ctrl_Input	36270757	35169739
SMS-CTRtetCASZ1_Dox_Input	40735455	39728585
SMS-CTRtetCASZ1_Ctrl_Ty1	41313225	37920917
SMS-CTRtetCASZ1_Dox_Ty1	35087452	33578246
SMS-CTRtetCASZ1_Ctrl_CASZ1	29847886	28892571
SMS-CTRtetCASZ1_Dox_CASZ1	33636741	32784786
SMS-CTRtetCASZ1_Ctrl_H3K27ac	30787681	30240045
SMS-CTRtetCASZ1_Dox_H3K27ac	38905126	38237253
SMS-CTRtetCASZ1_Ctrl_H3K27me3	31220731	28829049
SMS-CTRtetCASZ1_Dox_H3K27me3	28252454	26054645
SMS-CTRtetCASZ1_Ctrl_H3K4me3	39599825	38703301
SMS-CTRtetCASZ1_Dox_H3K4me3	39441714	38572536
SMS-CTRtetCASZ1_Ctrl_RNAPolII	38712772	37183917
SMS-CTRtetCASZ1_Dox_RNAPolII	41073978	39653442
SMS-CTR_Ctrl_Input	25030512	24303312
SMS-CTR_MEKi_Input	30289586	29290378
SMS-CTR_Ctrl_CASZ1	40942329	39574184
SMS-CTR_MEKi_CASZ1	45102854	43171454
C2C12_shCtrl_Input	30749936	29392134
C2C12_shCasz1_Input	30332148	28928788
C2C12_shCtrl_DM_H3K27ac	33885353	32762909
C2C12_shCasz1_DM_H3K27ac	36839736	35618082
C2C12_shCtrl_DM_H3K4me3	30315213	29141707
C2C12_shCasz1_DM_H3K4me3	31078266	29910441

Antibodies

CASZ1, home made by collaborating with Rockland Inc, affinity purified, named as Cas-ab8540
H3K27ac, Abcam, catalog number ab4729, lot number GR232896-1;
H3K27me3, EMD Millipore, catalog number 07-449, lot number 3083046;
H3K4me3, CST, catalog number 9751, lot number 10;
RNA polymerase II, Active Motif, catalog number 39097, lot number 30617017;
Ty1, Diagenode, catalog number C15200054, lot number 006

Peak calling parameters

Regions enriched in ChIP-seq signal were identified using MACS with corresponding control and parameters -f BAM -g mm -B -p 1e-7 -m 5 50 for narrowPeak calling and -f BAM --broad -g mm --broad-cutoff 0.1 -B -p 1e-7 -m 5 50 for broadPeak calling.

Data quality

Data quality was assessed using MACS by comparing peak enrichment over input controls with a p cutoff value of 1x10^-5 or 1x10^-7.

Software

Reads were aligned to the human genome (hg19) or mouse genome (mm10) using bwa mem with parameters -M -t 32. For visualization, tdf files were created from aligned ChIP-Seq read positions using igvtools count and to TDF with default parameters. Heatmaps of ChIP-Seq signal in these regions were made by using deeptools computeMatrix reference-point and plotHeatmap with default parameter (https://deeptools.readthedocs.io/en/develop/content/list_of_tools.html). Super-enhancers in were identified using ROSE (https://bitbucket.org/young_computation/rose) with default parameter.
